# The Cbs Locus Affects the Expression of Senescence Markers and mtDNA Copy Number, but not Telomere Dynamics in Mice

**DOI:** 10.3390/ijms21072520

**Published:** 2020-04-05

**Authors:** Olga Utyro, Joanna Perła-Kaján, Hieronim Jakubowski

**Affiliations:** 1 Department of Biochemistry and Biotechnology, University of Life Sciences, 60-632 Poznań, Poland; olga.utyro@gmail.com (O.U.); kajan@up.poznan.pl (J.P.-K.); 2 Institute of Bioorganic Chemistry, Polish Academy of Sciences, 61-704 Poznań, Poland; 3 Department of Microbiology, Biochemistry and Molecular Genetics, Rutgers-New Jersey Medical School, International Center for Public Health, Newark, NJ 07-103, USA

**Keywords:** cystathionine β-synthase deficiency, homocysteine, life span, mtDNA, senescence markers, telomere dynamics, Tert

## Abstract

Cystathionine β-synthase (CBS) is a housekeeping enzyme that catalyzes the first step of the homocysteine to cysteine transsulfuration pathway. Homozygous deletion of the *Cbs* gene in mice causes severe hyperhomocysteinemia and reduces life span. Here, we examined a possible involvement of senescence, mitochondrial DNA, and telomeres in the reduced life span of *Cbs*^−/−^ mice. We found that senescence-related *p21*, *Pai-1*, *Mcp1*, and *Il-6* mRNAs were significantly upregulated (2–10-fold) in liver, while *p21* was upregulated in the brain of *Cbs*^−/−^ mice (*n* = 20) compared with control *Cbs*^+/−^ siblings (*n* = 20) in a sex- and age-dependent manner. Telomere length in blood (*n* = 80), liver (*n* = 40), and brain (*n* = 40) was not affected by the *Cbs*^−/−^ genotype, but varied with sex and/or age. Levels of mitochondrial DNA tended to be reduced in livers, but not brains and blood, of *Cbs*^−/−^ females (*n* = 20–40). The *Cbs*^−/−^ genotype significantly reduced *Tert* mRNA expression in brain, but not liver, in a sex- and age-dependent manner. Multiple regression analysis showed that the senescence-related liver (but not brain) mRNAs and liver (but not brain or blood) mitochondrial DNA were associated with the *Cbs* genotype. In contrast, telomere length in blood, brain, and liver was not associated with the *Cbs* genotype or hyperhomocysteinemia, but was associated with sex (in brain and liver) and age (in brain and blood). Taken together, these findings suggest that the changes in senescence marker expression and mtDNA levels, but not telomere shortening, could account for the reduced life span of *Cbs*^−/−^ mice.

## 1. Introduction

Cellular senescence irreversibly arrests proliferation and is an important contributor to aging and age-related disease [1]. Senescence results in a loss of tissue repair ability due to stabilization of p53 and upregulation of the downstream p53 targets such as p21 and PAI-1 [2,3], which prevents CDK2-mediated inactivation of RB, thereby preventing entry into the S-phase of the cell cycle [1]. Senescent cells produce the senescence-associated secretory phenotype, i.e., pro-inflammatory and matrix degrading molecules (IL-6, MCP1, PAI-1, etc.). Although well studied in cultured cells in vitro, senescence in vivo in humans and animals is poorly understood [4]. One of the factors that induces the p53-p21 senescence pathway is telomere shortening [1].

Telomeres are tandem DNA repeats of TTAGGG structures at the end of chromosomes important for genomic stability in all vertebrates. The telomere sequence is more sensitive to damage than non-telomeric DNA, and its length progressively shortens with age during somatic cell division. Short telomeres are a feature associated with cellular senescence and death. Telomeres are synthesized by the telomerase (TERT) enzyme, a reverse transcriptase active during early human development, but becoming silenced in somatic cells at 12–18 weeks of gestation [5]. However, mice that have telomeres 50–150-kb long (5–10 times longer than humans, express Tert in somatic cells [6]. TERT activation occurs in 85–90% of all human cancers [7,8]. Short telomeres are usually found in a variety of human diseases that lead to premature death [9]. Thus, telomere length is considered to be a biomarker of aging and a major determinant of aging and the life span [6,10]. Recent findings in a variety of bird and mammalian species show that the telomere shortening rate, but not the telomere length alone, is a predictor of species life span [11]. Several genetic loci, including five known to be involved in telomere biology, are associated with telomere length and with increased risk of age-related diseases in humans [12]. Shorter telomeres are found in men vs. women, in older vs. younger individuals, and in Caucasian vs. other races [13].

Mitochondrial DNA (mtDNA) is an essential multicopy organellar genome organized into protein-DNA structures called nucleoids. The mtDNA lacks histones and is more sensitive to damage than nuclear DNA [14]. Like telomere length (TL), the mtDNA copy number changes with age [15]. A preponderance of evidence indicates that mtDNA is correlated with cellular aging and other age-related disorders such as cancer, diabetes, and neurodegenerative diseases [16,17]. Defects in mtDNA maintenance have been suggested to play a role in the mechanisms of aging [18]. However, a high mtDNA copy number is detrimental and leads to nucleoid enlargement, defective transcription, and age-related accumulation of mtDNA deletions, which causes respiratory chain protein deficiency [19].

Cystathionine β-synthase (CBS) deficiency, a rare metabolic disease caused by mutations in the *CBS* gene, is characterized by severely elevated levels of homocysteine (Hcy) and its metabolites [20,21,22,23] and pathologies in the cardiovascular, skeletal (osteoporosis), and nervous systems [24,25]. These pathologies are generally associated with aging. *CBS*^−/−^ patients are mentally retarded and experience thromboembolic events in brain, heart, and peripheral veins and arteries, which contribute to the reduced life span in these individuals [24,25].

Homozygous deletion of the *Cbs* gene leads to a neonatal lethality phenotype due to liver dysfunction with only a few percent of *Cbs*^−/−^ pups surviving to adulthood [26]. The creation of transgenic *Cbs*^−/−^ mice harboring wild-type or mutant versions of the human *CBS* gene under the control of the zinc-inducible metallothionein promoter prevents the neonatal lethality. One of those variants, the *Tg-I278T Cbs*^−/−^ mouse, has a median survival time 25% shorter compared to sibling controls (613 vs. 821 days) [27].

Although Cbs deficiency reduces life span, it is not known whether telomere shortening, senescence, and/or mtDNA copy number are involved. For this reason, the present work has been undertaken to study telomere length (TL), mtDNA levels, and *Tert* and senescence/aging marker (*Mcp1*, *Il-6*, *Pai-1*, *p21*, and klotho, *Kl*) mRNA expression in the tissues of severely hyperhomocysteinemic *Tg-I278T Cbs*^−/−^ mice and their sibling controls.

## 2. Results

### 2.1. Cbs Genotype Affects the Expression of Senescence-Related mRNAs in Mice

*Tg-I278T Cbs*^−/−^ mice have severe hyperhomocysteinemia (HHcy) (plasma total Hcy = 272 ± 50 μM compared to 5.0 ± 2.6 μM in *Cbs*^+/−^ siblings [28]), a 25% shorter life span (613 vs. 821 days) [27], and are characterized by a thin, smooth, shiny tail [29] and a reduced body weight [29]. Other characteristics of the *Tg-I278T Cbs*^−/−^ mice include facial alopecia, osteoporosis (rough periosteal surface and small holes in femur, reduced trabecular bone mass, decreased bone mineral density), and endoplasmic reticulum stress in liver and kidney [27]. These phenotypes are generally associated with aging.

**Figure 1:**
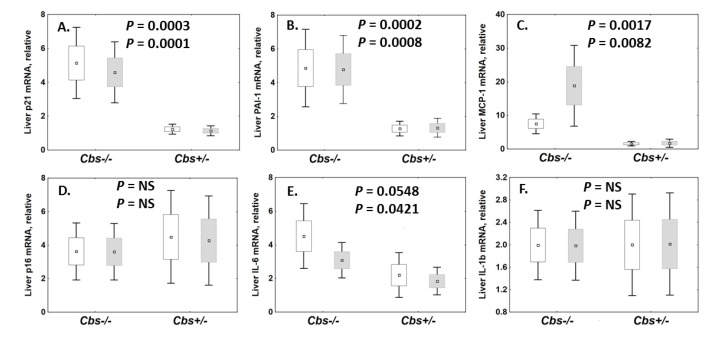
Box plots illustrating the effects of the *Cbs* genotype on the expression of senescence-related mRNAs in liver. The following mRNAs were quantified by qPCR in *Cbs*^−/−^ mice (*n* = 21) and sex- and age-matched control *Cb* ^+/−^ siblings (*n* = 22): **A**. *p21*, **B**. *Pai-1*, **C**. *Mcp-1*, **D**. *p16*, **E**. *Il-6*, and **F**. *Il-1β*. Box and whiskers represent the mean ± SEM and the mean ± 95% CI, respectively. Upper and lower *p*-values refer to data normalized to β-actin (blank boxes) and Gapdh (gray boxes) mRNAs, respectively. NS, not significant.

We hypothesized that the reduced life span of the *Cbs*^−/−^ mice could be caused by premature senescence. To examine this hypothesis, we quantified the expression of senescence-related mRNAs (*Pai-1*, *p21*, *Mcp1*, *Il-6*, *p16*, and *Il-1β*) and an aging-related *Kl* mRNA relative to β-*actin* and *Gapdh* mRNAs in mouse brains and livers. We found that *Pai-1*, *p21*, *Mcp1*, and *Il-6* mRNAs were significantly upregulated in livers of *Cbs*^−/−^ mice compared to *Cbs*^+/−^ siblings (Figure 1A–C,E), while *p16* and *Il-1β* mRNAs were not affected by the *Cbs*^−/−^ genotype (Figure 1D,F). The expression of the corresponding mRNAs in brains was similar between *Cbs*^−/−^ mice and their *Cbs*^+/−^ siblings (not shown).

#### 2.1.1. Liver

We also found that the effects of the *Cbs*^−/−^ genotype on the expression of senescence-related mRNAs were sex- and age-dependent. In young *Cbs*^−/−^ female mice, liver *p21*, *Pai-1*, and *Mcp-*1 mRNAs were significantly upregulated (7.6- to 11.0-fold, *p* = 0.014–0.025) compared to young *Cbs*^+/−^ females. Liver *p21* and *Pai-1* mRNAs were also upregulated (4.2- to 4.4-fold, *p* = 0.006–0.059) in old *Cbs*^−/−^ females (Supplementary Table S1).

In young *Cbs*^−/−^ male mice, liver *p21*, *Mcp-1*, and *Il-6* mRNAs were upregulated (3.0- to 8.7-fold, *p* = 0.005–0.063) compared to young *Cbs*^+/−^ males. Liver *Mcp-1* mRNA was also upregulated (3.2-fold, *p* = 0.034) in old *Cbs*^−/−^ males (Supplementary Table S1).

Sex significantly affected the expression of liver *Mcp-1* (lower in *Cbs*^−/−^ males, *p* = 0.004) and *Il-6* mRNAs (higher in *Cbs*^+/−^ males, *p* = 0.010) (Supplementary Table S1).

In *Cbs*^−/−^ mice, age significantly affected the expression of liver *Pai-1* (lower in old *Cbs*^−/−^ females, *p* = 0.004) and *Mcp-1* mRNAs (higher in old *Cbs*^−/−^ males, *p* = 0.034). In *Cbs*^+/−^ mice, age affected the expression of liver *Mcp-1*, *Il-6* (higher in old *Cbs*^+/-^ females and males) and *p21* mRNAs (higher in old *Cbs*^+/-^ females) (Supplementary Table S1).

Although the *Cbs* genotype had no effect on *Il-1β* and *p16* mRNAs in the whole mouse cohort (Figure 1D,F), subgroup analyses showed that *p16* mRNA was downregulated in young and old female *Cbs*^−/−^ mice relative to *Cbs*^+/−^ females (young: 0.04 vs. 0.15, *p* = 0.035; old: 6.5 vs. 14.1, *p* = 0.046), but not in males. *Il-1β* mRNA was not affected by the *Cbs* genotype in any of the age and sex subgroups. *Il-1β* mRNA was significantly upregulated in old mice, similarly in *Cbs*^−/−^ and *Cbs*^+/−^ females and males (2.5- to five-fold; *p* = 0.015–0.039).

#### 2.1.2. Brain

The expression of senescence-related brain *p21*, *Mcp-1*, *Pai-1*, and *Il-6* mRNAs appeared to be unaffected by the *Cbs*^−/−^ genotype in the whole mouse cohort, as was Kl mRNA (not shown). However, examination of sex and age subgroups revealed that, in *Cbs*^−/−^ female mice, brain *p21* and *Mcp-1* mRNAs were significantly upregulated relative to *Cbs*^+/−^ females (1.9-fold in young, *p* = 0.035, and 1.4-fold in old females, *p* = 0.020, respectively; Supplementary Table S2). However, the expression of the senescence-related brain mRNAs was unaffected by the *Cbs*^−/−^ genotype in males.

Sex significantly affected the expression of brain *p21*, *Mcp-1*, *Il-6*, and *Kl* mRNAs in *Cbs*^−/−^ mice, (1.4- to 2.5-fold higher in females than in males, *p* = 0.040 to 0.004). In *Cbs*^+/−^ mice, sex affected the expression of brain *p21* in old mice (2.1-fold higher in females, *p* = 0.036) and *Mcp-1* mRNAs (lower in females, *p* = 0.052; Supplementary Table S2).

Age significantly affected the expression of brain *Mcp-1* (1.6-fold higher in old vs. young *Cbs*^−/−^ females, *p* = 0.020), *Kl* (0.6-fold lower in old *Cbs*^−/−^ females, *p* = 0.022), and *p21* (0.5-fold lower in old vs. young *Cbs*^+/−^ males, *p* = 0.038) mRNAs (Supplementary Table S2).

Brain *Il-1β* and *p16* mRNAs were not affected by the *Cbs* genotype in any of the age and sex subgroups. Age and sex did not affect brain *Il-1β* mRNA, while age significantly affected brain *p16* mRNA (six- to 10-fold higher in old vs. young mice, *p* = 0.009 to 0.000).

### 2.2. Cbs Genotype and TL

#### 2.2.1. Blood TL

**Figure 2:**
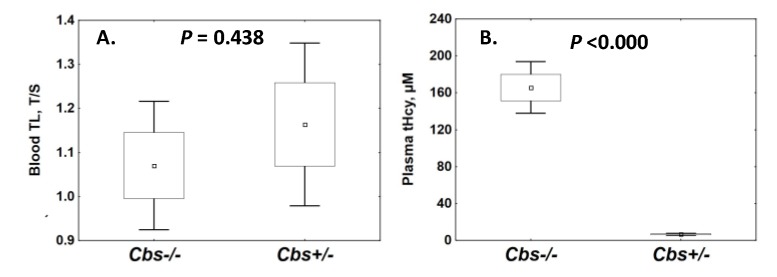
Blood telomere length (TL) (**A**) and plasma total Hcy (tHcy) levels (**B**) in *Cbs*^−/−^ and *Cbs*^+/−^ mice. Blood TL and plasma tHcy were quantified in *Cbs*^−/−^ mice (*n* = 40) and sex- and age-matched control *Cbs*^+/−^ siblings (*n* = 40) as described in the Materials and Methods. Box and whiskers represent the mean±SEM and the mean ± 95% CI, respectively. Upper and lower *p*-values refer to data normalized to β-actin (blank boxes) and Gapdh (gray boxes) mRNAs, respectively.

We quantified TL in the blood of *Cbs*^−/−^ mice (*n* = 40) and their *Cbs*^+/−^ siblings (*n* = 40) and found that blood TL was not affected by the *Cbs* genotype (Figure 2A). In males, blood TL decreased with age over the span from 36 to 473 days, similarly in both *Cbs*^−/−^ mice and their *Cbs*^+/−^ siblings (Supplementary Figure S1A). In females, blood TL was also similar between *Cbs*^−/−^ and *Cbs*^+/−^ mice, and there was little change in blood TL with age (Supplementary Figure S1B). Blood TL tended to decrease with increasing plasma tHcy in male, but not in female mice (Supplementary Figure S1C).

Further analyses showed that blood TL was similar between *Cbs*^−/−^ and *Cbs*^+/−^ young females (1.08 ± 0.31 vs. 1.12 ± 0.41, *p* = 0.820) and males (1.19 ± 0.27 vs. 1.34 ± 0.27, *p* = 0.335). Blood TL was also similar between old *Cbs*^−/−^ and *Cbs*^+/−^ mice of both sexes (females: 1.10 ± 0.19 and 1.01 ± 0.18, *p* = 0.182; males: 0.68 ± 0.17 and 0.64 ± 0.19, *p* = 0.615) (Supplementary Table S3).

Total Hcy levels were severely elevated in *Cbs*^−/−^ compared to *Cbs*^+/−^ mice (166 ± 80 μM vs. 6.6 ± 2.9 μM, *p* < 0.000) (Figure 2B), as expected [27,28]. Levels of tHcy increased with age in *Cbs*^−/−^ females (238 ± 33 vs. 133 ± 64 μM, *p* = 0.002), but not in males, and were significantly higher in females compared with males (238 ± 33 vs. 156 ± 96 μM, *p* = 0.044) (Supplementary Table S3). Notably, in the whole mouse cohort (Figure 1A) and in sex- and age-stratified groups (Supplementary Table S3), blood TL was not significantly different between severely HHcy *Cbs*^−/−^ mice compared to *Cbs*^+/−^ siblings, which had normal tHcy levels.

##### Effects of Age on Blood TL in *Cbs*^+/−^ and *Cbs*^−/−^ Mice

In male *Cbs*^−/−^ mice, blood TL was significantly reduced in older compared to younger animals (0.68 ± 0.17 vs. 1.19 ± 0.27, *p* < 0.000) (Supplementary Table S3). Similar reductions in older vs. younger mice were also observed in *Cbs*^+/−^ males (0.64 ± 0.19 vs. 1.34 ± 0.27, *p* < 0.000) (Supplementary Table S3). In contrast, in female *Cbs*^−/−^ and *Cbs*^+/−^ mice, age did not affect blood TL (Supplementary Table S3).

##### Effects of Sex on Blood TL in *Cbs*^+/−^ and *Cbs*^−/−^ Mice

The effect of sex on blood TL was similar between *Cbs*^+/−^ and *Cbs*^−/−^ mice. Specifically, blood TL was significantly higher in old *Cbs*^+/−^ and *Cbs*^−/−^ females compared with old males (*Cbs*^+/−^: 1.01 ± 0.18 vs. 0.64 ± 0.19, *p* < 0.000; *Cbs*^−/−^: 1.10 ± 0.19 vs. 0.68 ± 0.17, *p* < 0.000) (Supplementary Table S3). However, this sex-specific difference in TL was absent in young *Cbs*^+/−^ and *Cbs*^−/−^ mice.

#### 2.2.2. Brain TL and *Tert* mRNA

To examine whether *Cbs* genotype affects TL in a tissue-specific manner, we isolated DNA from brains and livers of *Cbs*^−/−^ mice (*n* = 19) and their *Cbs*^+/−^ siblings (*n* = 24) and quantified brain and liver TL. To examine whether TL is affected by telomerase expression, we isolated RNA and examined *Tert* mRNA levels in these tissues.

We found that brain TL was not affected by the *Cbs* genotype (Supplementary Figure S2A). However, brain TL was affected by sex in a *Cbs* genotype- and an age-dependent manner. Specifically, in old, but not young, *Cbs*^−/−^ mice, brain telomeres were significantly longer in males compared to females (1.53 ± 0.34 vs. 0.95 ± 0.22, *p* = 0.010). In contrast, in young and old *Cbs*^+/−^ mice, brain TL was not affected by sex and was similar between males and females (Supplementary Table S4).

We also found that brain *Tert* mRNA expression was not affected by the *Cbs* genotype in the whole mouse cohort (Supplementary Figure S3A). However, *Tert* mRNA appeared to be affected by the *Cbs* genotype in some age-and sex-stratified subgroups. Specifically, young, but not old, *Cbs*^−/−^ male, but not female, mice had significantly reduced expression of brain *Tert* mRNA compared to *Cbs*^+/−^ animals (0.55 ± 0.17 vs. 1.26 ± 0.39, *p* = 0.035) (Supplementary Table S4).

Brain *Tert* mRNA expression was also affected by sex. Specifically, in young *Cbs*^−/−^ mice, brain *Tert* mRNA was significantly elevated in females compared to males (1.29 ± 0.33 vs. 0.55 ± 0.17, *p* = 0.017) (Supplementary Table S4). In old *Cbs*^+/−^ mice, brain *Tert* mRNA tended to be reduced in males compared to females (0.66 ± 0.21 vs. 1.28 ± 0.61, *p* = 0.067).

Brain *Tert* mRNA expression was also affected by age in a sex-dependent manner. Specifically, brain *Tert* mRNA levels were significantly reduced in old compared to young male (0.66 ± 0.21 vs. 1.26 ± 0.39, *p* = 0.022), but not female, *Cbs*^+/−^ mice; however, this effect of age was absent in *Cbs*^−/−^ mice (Supplementary Table S4).

#### 2.2.3. Liver TL and *Tert* mRNA

Liver TL, similar to blood TL (Figure 2, Supplementary Table S3) and brain TL (Supplementary Figure S2A), was not affected by the *Cbs* genotype (Supplementary Figure S2B). However, liver TL was affected by sex in an age-dependent manner. For example, in old mice, liver TL were significantly longer in females compared to males, both in *Cbs*^−/−^ (1.11 ± 0.30 vs. 0.75 ± 0.19, *p* = 0.014) and *Cbs*^+/−^ mice (1.50 ± 0.58 vs. 0.78 ± 0.23, *p* = 0.018) (Supplementary Table S5). In contrast, in young *Cbs*^−/−^ and *Cbs*^+/−^ mice, there was no significant difference in liver TL between females and males (Supplementary Table S5).

Similar to brain TL (Supplementary Table S4), but in contrast to blood TL (Figure 2, Supplementary Table S3), liver TL was not affected by age in *Cbs*^−/−^ and *Cbs*^+/−^ mice (Supplementary Table S5).

Liver *Tert* mRNA expression was also similar between *Cbs*^−/−^ and *Cbs*^+/−^ mice (Supplementary Figure S3B), but was affected by sex in a *Cbs* genotype- and age-dependent manner. Specifically, in young *Cbs*^−/−^ mice, liver *Tert* mRNA was lower in females compared to males (0.22 ± 0.10 vs. 0.43 ± 0.03, *p* = 0.020) (Supplementary Table S5). In old *Cbs*^−/−^ mice, liver *Tert* mRNA expression was lower in females compared to males (2.22 ± 0.21 vs. 1.43 ± 0.47, *p* = 0.029). In old *Cbs*^+/−^ mice, liver *Tert* mRNA was higher in females compared to males (2.89 ± 1.07 vs. 1.43 ± 0.39, *p* = 0.012) (Supplementary Table S5).

### 2.3. Cbs Genotype and mtDNA Copy Number in Blood, Liver, and Brain

In the whole mouse cohort, blood, liver, and brain mtDNA levels were not affected by the Cbs genotype (Supplementary Figure S4). However, mtDNA was affected by age and sex (see below).

#### 2.3.1. Blood mtDNA

**Figure 3:**
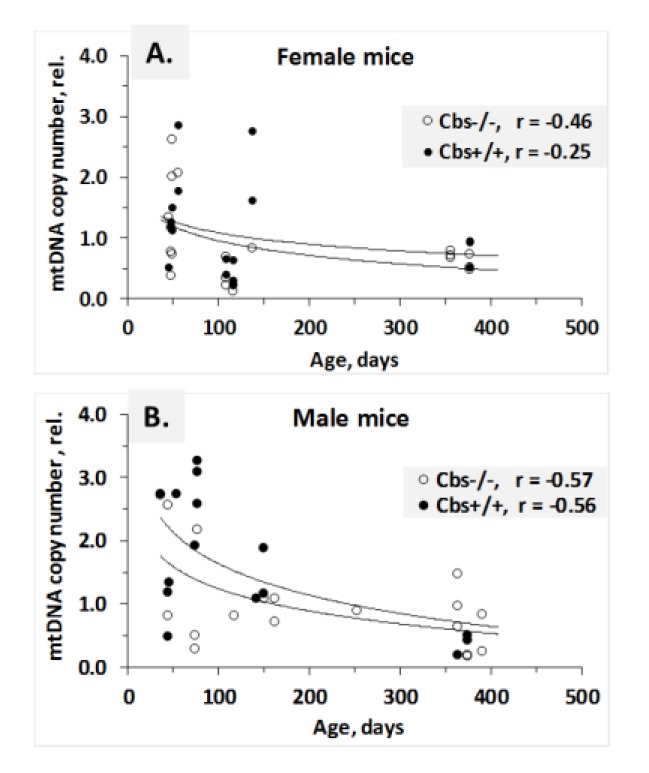
Relationships between blood mtDNA copy number and age in female (**A**) and male (**B**) *Cbs*^−/−^ and *Cbs*^+/−^ mice. ♀ *Cbs*^−/−^ (*n* = 11), ♂ *Cbs*^−/−^ (*n* = 10), ♀ *Cbs*^+/−^ (*n* = 10), and ♂ *Cbs*^+/−^ (*n* = 13). Logarithmic trendlines fitted to the data points are shown. rel. = relative.

Relationships between blood mtDNA and age for *Cbs*^−/−^ and *Cbs*^+/−^ mice of each sex are shown in Figure 3. Overall, blood mtDNA decreased with age over the span from 36 to 390 days, similarly for *Cbs*^−/−^ and *Cbs*^+/−^ mice and for both females and males.

Further analyses showed that blood mtDNA was not affected by the *Cbs* genotype, but was affected by sex and age: higher in young compared with old female *Cbs*^−/−^ (1.39 ± 0.78 vs. 0.68 ± 0.13, *p* = 0.004) and *Cbs*^+/−^ (1.46 ± 0.72 vs. 0.84 ± 0.75, *p* = 0.103) mice. mtDNA was also higher in young vs. old male *Cbs*^−/−^ (1.51 ± 1.09 vs. 0.76 ± 0.40, *p* = 0.046) and *Cbs*^+/−^ (2.15 ± 0.96 vs. 0.87 ± 0.62, *p* = 0.003) mice (Supplementary Table S6).

Blood mtDNA was also affected by sex in young, but not old, *Cbs*^+/−^ mice: higher in young males compared with young females (2.15 ± 0.96 vs. 1.46 ± 0.72, *p* = 0.041). This sex effect was abrogated by the *Cbs*^−/−^ genotype (1.51 ± 1.09 vs. 1.39 ± 0.78, *p* = 0.322) (Supplementary Table S6).

#### 2.3.2. Brain and Liver mtDNA

Liver mtDNA tended to decrease in *Cbs*^−/−^ mice compared to *Cbs*^+/−^ animals (1.12 ± 0.49 vs. 1.75 ± 0.53, *p* = 0.059), while brain mtDNA was not affected by the *Cbs* genotype (Supplementary Table S6).

We found that brain and liver mtDNAs were affected by sex (higher in males) and age (Supplementary Table S6). However, in contrast to blood mtDNA, which decreased with age, mtDNA significantly increased in brains of old compared to young *Cbs*^−/−^ males (1.31±0.23 vs. 0.95 ± 0.14, *p* = 0.002) and *Cbs*^+/−^ males (1.40 ± 0.60 vs. 0.89 ± 0.13, *p* = 0.048). Increased mtDNA levels were also found in livers of old compared to young *Cbs*^−/−^ and *Cbs*^+/−^ mice of both sexes.

### 2.4. Determinants of Blood TL in Mice

Associations between blood TL and independent variables are shown in Table 1 and Table 2. Pearson correlation analyses of a whole cohort including *Cbs*^−/−^ and *Cbs*^+/−^ mice showed that blood TL was significantly associated with age, but not with the *Cbs* genotype or tHcy (Table 1).

Multiple regression analysis for the whole cohort including *Cbs*^−/−^ and *Cbs*^+/−^ mice of both sexes showed that blood TL was not associated with the *Cbs* genotype, plasma tHcy, age, or sex (Table 1). However, in male mice, blood TL was negatively associated with age, which explained 18–34% of the TL variation (Table 1).

### 2.5. Determinants of TL in Mouse Brain and Liver

Comparative multiple regression analysis of determinants of blood TL, brain TL, and liver TL is shown in Table 2. Similar to blood TL, brain and liver TL were also found not to be associated with the *Cbs* genotype. However, in contrast to blood TL, brain TL was associated with sex and age, while liver TL was associated with sex, explaining 39% and 26% of the TL variation in brain and liver, respectively (Table 2).

### 2.6. Determinants of Tert mRNA Expression in Mouse Brain and Liver

Multiple regression analysis in a model involving sex, age, the *Cbs* genotype, and TL showed that the brain and liver *Tert* mRNAs were not associated with the *Cbs* genotype (Supplementary Table S7). However, brain *Tert* mRNA expression was associated with sex (Supplementary Table S7), young *Cbs*^−/−^ female mice having higher expression than males (1.29 ± 0.33 vs. 0.55 ± 0.17, *p* = 0.017, Supplementary Table S4), explaining 28% of the variability in *Tert* mRNA (Supplementary Table S7). Liver *Tert* mRNA was positively associated with TL and age, which explained 69% variability in *Tert* mRNA expression (Supplementary Table S7).

### 2.7. Determinants of Blood, Brain, and Liver mtDNA

#### 2.7.1. Pearson Correlations

Pearson correlation analysis of the whole cohort including *Cbs*^−/−^ and *Cbs*^+/−^ mice of both sexes showed that blood mtDNA was associated with the *Cbs* genotype (*p* = 0.044), sex (*p* = 0.021), and age (*p* = 0.025) (Table 3). Brain mtDNA was associated with sex (*p* = 0.007) and age (*p* = 0.005). Liver mtDNA was associated with age.

Pearson correlation analysis stratified by sex showed that blood mtDNA was significantly associated with the *Cbs* genotype (*p* = 0.014) and age (*p* = 0.005) in males. Brain and liver TL were associated with age in males and females.

#### 2.7.2. Multiple Regression

In multiple regression analysis for the whole mouse cohort in models including sex, age, and the *Cbs* genotype or plasma tHcy, liver mtDNA was significantly associated with the *Cbs* genotype (β = 0.24, *p* = 0.041) and age (β = 0.70, *p* = 0.000) in the whole mouse cohort including both sexes. The association with the *Cbs* genotype was somewhat stronger in the female subgroup (β = 0.35, *p* = 0.030). In contrast, in the male subgroup, age (*p* = 0.003), but not the *Cbs* genotype, was the only significant determinant of liver mtDNA. The *Cbs* genotype and age explained 44% of the liver mtDNA variability in the whole cohort and 63 % in the female subgroup (Table 3).

In contrast, brain and blood mtDNAs were not associated with the *Cbs* genotype, but were associated with sex and age, which explained 35% (brain) and 24–29% (blood) of the mtDNA variability (Table 3). Brain mtDNA was also significantly associated with age in the female (β = 0.49, *p* = 0.023) and male (β = 0.49, *p* = 0.032) subgroups.

Multiple regression analysis of subgroups in models involving age and the *Cbs* genotype or plasma tHcy showed that most of the blood mtDNA variability occurred in males (R^2^ = 0.35–0.48), not females (R^2^ = 0.01–0.1). The blood mtDNA was strongly associated with age in males (β = −0.58, *p* = 0.000) and tended to be associated with the *Cbs* genotype (*p* = 0.058), but not plasma tHcy (*p* = 0.419). Age and the *Cbs* genotype explained 48% of the variability in mtDNA in males (Table 3).

### 2.8. Determinants of Senescence-Related mRNAs in Mice

Associations between senescence-related mRNAs and independent variables in mouse brain and liver are presented in Table 4.

#### 2.8.1. Liver

Multiple regression analysis for the whole cohort of mice in models including sex, age, the *Cbs* genotype, TL, *Tert* mRNA, and mtDNA showed that the *Cbs* genotype was the strongest determinant of the liver *Pai-1*, *p21*, and *Mcp1* mRNAs (*p* = 0.001), while liver mtDNA was the strongest determinant of liver *Il-6* (*p* = 0.000, Table 4) and *Il-1β* mRNAs (*p* = 0.000, Table 5).

Multiple regression analysis for the female mice subgroup showed that the *Cbs*^−/−^ genotype was the strongest positive determinant of liver *Pai-1*, *p21*, *Mcp1*, and *Il-6* mRNAs (*p* = 0.021–0.007). In addition, *Tert* mRNA (*p* = 0.002–0.006) was a positive determinant of liver *Pai-1*, *Mcp1*, and *Il-6* mRNAs, while liver mtDNA (*p* = 0.002) was a positive determinant of liver *Pai-1* and *Il-6* mRNAs in females (Table 4). The *Cbs* genotype tended to be associated with liver *p16* mRNA (*p* = 0.086, Table 5).

In the male mice subgroup, the *Cbs*^−/−^ genotype was a significant positive determinant of liver *p21* (*p* = 0.034) and *Mcp1* (*p* = 0.013) mRNAs. In addition, liver *Tert* mRNA (*p* = 0.020) and age (*p* = 0.002) were positive determinants of liver *Pai-1* and *Mcp1* mRNAs, respectively; liver *Tert* mRNA (*p* = 0.016) was a negative determinant, while liver mtDNA (*p* = 0.001) and age (*p* = 0.015) were positive determinants of liver *Il-6* mRNA (Table 4).

#### 2.8.2. Brain

Multiple regression analysis for the whole cohort of mice showed that the *Cbs* genotype was not a determinant of any senescence-related mRNAs in brain (Table 4).

However, multiple regression analysis identified other brain variables as significant positive determinants of brain senescence-related mRNAs: mtDNA (*p* = 0.018) of *Pai-1* mRNA, sex (*p* = 0.044), and TL (*p* = 0.028) of *p21* mRNA (higher in females) and age (*p* = 0.008) and *Tert* mRNA (*p* = 0.019) of *Il-6* and *Kl* mRNAs, respectively (Table 4). Age and sex were determinants of *p16* mRNA (*p* = 0.001, Table 5).

In multiple regression analysis for sex subgroups, age was a positive determinant of brain *Mcp1* and *Il-6* mRNAs in male mice and a negative determinant of brain *Kl* mRNA in female mice (Table 4). The *Cbs* genotype tended to be associated with brain *p16* mRNA (*p* = 0.065, Table 5).

## 3. Discussion

Cbs deficiency has been known to reduce life span both in humans and mice. However, the underlying mechanism of the reduced life span in Cbs deficiency was unknown. In the present work, we examined a possible involvement of telomere shortening, mtDNA copy number, and accelerated senescence in the reduced life span of *Cbs*^−/−^ mice.

We used a *Cbs*^−/−^ mouse model that recapitulated the reduced life span and pathological phenotypes generally associated with aging observed in CBS-deficient patients. We found that the expression of senescence-associated mRNAs (two targets of p53: *Pai-1* and *p21*; and three components of the senescence-associated secretory phenotype: *Mcp-1*, *Pai-1*, and *Il-6*) were significantly upregulated in livers of *Cbs*^−/−^ mice compared to control *Cbs*^+/−^ siblings (Figure 1). However, the expression of liver *p16* and *Il-1β* and the antiaging Kl protein mRNAs was not affected by the *Cbs* genotype. These expression patterns were specific to liver and the *Cbs*^−/−^ genotype had no effect on the expression of any of these mRNAs in brain. It should be noted that *p16* mRNA, which is considered to be a more specific marker for senescence, is not expressed by all senescent cells [30] and is expressed in certain non-senescent cells [31]. The senescence phenotype is known to be variable [4,30,31], and our findings in *Cbs*^−/−^ mice provided another example of such variability.

The present findings suggested that accelerated senescence could provide a plausible molecular mechanism contributing to hepatic steatosis observed in *Cbs*^−/−^ mice [26,27,32]. Importantly, our findings also suggested that accelerated senescence in liver could also explained the reduced life span associated with *Cbs* deficiency in mice.

We found that the *Cbs* genotype and HHcy did not affect TL in blood (Figure 2), brain, and liver (Supplementary Figure S2). We also found that *Tert* mRNA expression was not associated with the *Cbs* genotype, but was associated with TL in liver and not in brain. The lack of association of TL with the *Cbs* genotype suggested that the reduced life span in *Cbs*^−/−^ mice was caused by accelerated senescence in liver and was unlikely to be mediated by HHcy or telomere shortening.

Although we found no association between the *Cbs* genotype and TL in any of the threes mouse tissues that were examined, we did find that age and sex, known to affect TL in other biological systems [4,5], were associated with TL in our mouse cohort (Table 1 and Table 2). For example, blood TL was associated with age in male mice and brain TL with sex and age, while liver TL was associated with sex. These effects of age/sex on TL provided positive controls and indicated that our assays were sufficiently sensitive to allow identification of variables known to be associated with TL, thereby increasing confidence in the present findings.

We also found that the *Cbs* genotype was a significant determinant of liver mtDNA copy number (Table 3). Specifically, liver mtDNA copy number was reduced in *Cbs*^−/−^ mice compared with *Cbs*^+/−^ sibling controls, suggesting the involvement of hepatic respiratory chain deficiency in the reduced life span of *Cbs*^−/−^ mice. In contrast, the *Cbs* genotype did not affect the brain mtDNA copy number, showing that the *Cbs*^−/−^ genotype-induced reduction in mtDNA copy number was liver specific. Because mtDNA copy number is essential for maintaining oxidative capacity, ATP generation, and ultimately cell survival, our findings suggested that reduced liver mtDNA copy number could be responsible for the reduced life span in *Cbs*^−/−^ mice.

Our findings also showed that mtDNA copy number in *Cbs*^−/−^ and *Cbs*^+/−^ mice changed with age in a tissue-dependent manner: decreases in blood and increases in brain and liver (Table 3; Supplementary Table S6). Similar tissue-dependent changes have been previously reported in aging wild-type mice [15,33] and rats [34] by other investigators. It should be noted, however, that both decreased and increased copy number of mtDNA can cause mitochondrial dysfunction [19]. In fact, increased mtDNA copy number is found in human patients with bipolar disorder, which is associated with accelerated aging [35]. However, similar to the present findings in *Cbs*-deficient mice, TL was not affected in the bipolar disorder patients.

The limitations of this study should be noted. First, we did not study the expression of senescence-associated genes at the protein level. Measuring senescence-related protein levels in the mouse (e.g., p16) can be challenging mostly due to the availability of antibodies [36]. However, as regulation of these genes occurs at the transcriptional level [4,30,31], one can expect to observe similar changes at the protein level caused by the *Cbs* genotype. Second, we did not have tissues suitable for immunohistochemistry of markers such as senescence-associated β-galactosidase (SA-β-gal). However, although SA-β-gal is prominent in senescent sells, it is neither required, nor a determinant of the senescence phenotype [4,37]. Third, although the number of *Cbs*^−/−^ and *Cbs*^−/−^ mice was 20 to 40 per each *Cbs* genotype group, the number of animals in the sex- and age-stratified subgroups was accordingly much lower. However, despite the relatively low number of animals in these subgroups, we found that age and sex, which are known to affect TL [13,15], were associated with TL in the present study (Table 1 and Table 2). We also found that age, known to affect mtDNA copy number in wild-type mice [15,33] and rats [34], was associated with mtDNA in the present study (Figure 3 and Table 3).

In conclusion, we showed that *Cbs*^−/−^ mice had significantly upregulated expression of some senescence-related mRNAs (*Pai-1*, *p21*, *Mcp-1*, and *Il-6*) in liver, but not in brain, and reduced mtDNA copy number in liver, but not in brain and blood. We also showed that TL in any of those tissues was not affected by the *Cbs*^−/−^ genotype. These findings suggested that accelerated senescence, mostly affecting liver, and impaired mitochondrial function, but not telomere shortening, could contribute to the reduced life span of *Cbs*^−/−^ mice.

## 4. Materials and Methods

### 4.1. Mice

Transgenic *Tg-I278T Cbs*^−/−^ mice on a C57BL/6J genetic background [27] were mated with their *Tg-I278T Cbs*^+/-^ counterparts to generate sufficient numbers of *Tg-I278T Cbs*^−/−^ and *Tg-I278T Cbs*^+/−^ animals required for the experiments. The mice were bred and housed at the New Jersey Medical School Animal Facility [29,38]. In these animals, the human *CBS-I278T* variant was under the control of the zinc-inducible metallothionein promoter, which prevents the neonatal lethality of the mouse *Cbs*^−/−^ genotype by supplementation of the drinking water with 25 mM ZnCl_2_ [27]. The zinc water was replaced by plain water after weaning at 30 days. The mice were fed a normal rodent chow (LabDiet5010, Purina Mills International, St. Louis, MO, USA). The *Cbs* genotype was established by PCR using the following primers: forward 5’-GGTCTGGAATTCACTATGTAGC-3’, *Cbs*- reverse 5’- GAGGTCGACGGTATCGATA-3’ (affording a 176 bp product), *Cbs*+ reverse 5’-CGGATGACCTGCATTCATCT-3’ (affording a 300 bp product). Mice (*n* = 40 to 80) of both sexes, 63 to 408 days old, were used in experiments. Animal procedures were approved by the Institutional Animal Care and Use Committee at the New Jersey Medical School.

### 4.2. Blood and Tissue Collection

Blood was collected from cheek veins into Eppendorf tubes containing 1% (*v/v*) 0.5 M EDTA. After centrifugation (2000× *g*, 10 min, 4 °C), separated plasma and cells were frozen at −80 °C. Mice were euthanized using CO_2_ and the organs collected and frozen on dry ice. Brains and livers were powdered with dry ice using a mortar and pestle and stored at −80 °C.

### 4.3. Total Hcy Assays

Plasma tHcy was quantified by the conversion to Hcy-thiolactone, which was then separated by cation exchange HPLC using a Poly CAT A column, 35 × 2.1 mm, 5 µM, 300 Å (PolyLC), post-column derivatized with *o*-phthalaldehyde, detected and quantified by fluorescence as previously described [28,39]. An Agilent Infinity 1260 system, containing a degasser, binary pump, high performance auto-sampler, thermostated column compartment, and fluorescence detector, was used.

### 4.4. DNA Extraction

Total DNA, extracted from brain, liver, or whole mouse blood using the phenol method, was treated with RNase A (Thermo Scientific, Warsaw, Poland) and diluted with ultrapure water. The integrity and quality of the DNA were tested by agarose gel electrophoresis and spectroscopy (Thermo Scientific, Warsaw, Poland).

### 4.5. Telomere Length Analysis

Mouse genomic DNA was extracted from the whole blood using the phenol extraction method and stored at −80 °C. The telomere length assays were performed using the quantitative polymerase chain reaction (PCR) method to measure telomere length relative to standard reference DNA (T/S ratio), as described in detail elsewhere [40,41]. The telomeres were amplified using primers specific for the telomere repeats (forward: 5’-ACACTAA(GGTTTG)_4_GGTTAGTGT-3’; reverse 5’-TGTTAGG-(TATCCC)_5_TAACA-3’). The amplification of the single copy gene albumin (forward: 5’-CGGCG-GCGGGCGGCGCGGGCTGGGCGGAAGTGCTGTAGTGGATCC-CTG-3’; reverse: 5’-GCCCGGCC-CGCCGCGCCCGTCCCGCCGGAGAAGCATGGCCGCCTTT-3’) was used as a control for the input DNA [40,41].

The master mix for each PCR amplification tube was prepared with iTaq™ Universal SYBR^®^ Green Supermix (Bio-Rad Polska Sp. z o.o., Warsaw, Poland), 900 nM of telomere- or albumin-specific primers, and 160 ng DNA as a template. The optimized thermal cycling profile for each reaction mixture was as follows: Step 1: 94 °C for 15 min.; Step 2: 94 °C for 15 s, 49 °C for 15 s, two cycles; Step 3: 94 °C for 15 s, 62 °C for 10 s, 74 °C for 15 s with signal acquisition, 84 °C for 10 s, 88 °C 15 s with signal acquisition, 35 cycles; Step 4: 65–95 °C with signal acquisition (melting curve analysis). The 74 °C reads provided the cycle threshold (*C*t) values for the amplification of the telomere template, and the 88 °C reads provided the *C*t values for the amplification of the albumin. All qPCRs were carried out on a CFX96 Touch™ Real-Time PCR Detection System (Bio-Rad Polska Sp. Z o.o.).

The relative telomere length (TL) was calculated from the Ct values as the ratio of the telomere signal to the single copy albumin gene signal (T/S ratio) [40,41]. To calculate Δ*C*t, the Ct for the single-copy albumin gene was subtracted from the telomere Ct (Δ*C*t = *C*t_telomere_ − *C*t_albumin_). To calculate ΔΔ*C*t, the Δ*C*t for the DNA from control *Cbs*^+/−^ individuals was averaged and subtracted from the Δ*C*t for the DNA from each *Cbs*^−/−^ mouse and *Cbs*^+/−^ control animal (ΔΔ*C*t = Δ*C*t*_Cbs_*_−/−_ − Δ*C*t*_Cbs_*_+/−_). The T/S values were calculated according to the T/S = 2^−(^^ΔΔ*C*t)^ equation [42]. Each sample was measured in duplicate. The reproducibility of the measurements was 6%.

### 4.6. mtDNA Quantification

DNA was diluted with ultrapure water and sonicated in an ultrasonic bath (45 kHz, 5 min) prior to qPCR analysis [43]. The mtDNA was amplified using the following primers: forward 5’-CTAGAAACCCCGAAACCAAA-3’, reverse 5’-CCA-GCTATCACCAAGCTCGT-3’. The amplification of the single copy nuclear gene β2 microglobulin (B2M) (forward 5’-ATGGGAAGCCGAACATACTG-3’, reverse 5’-CAGTCTCAGTGGGGGTGAAT-3’) was used for normalization of mtDNA.

All reactions were performed in 10 µL volumes in duplicate using 20 ng DNA, 3 µM primers, and 5 µL iTaq Universal SYBR Green Supermix (Bio-Rad Polska Sp.z o.o.) using a CFX96 Touch Real Time PCR Detection System (Bio-Rad). Cycling parameters: 95 °C (10 min), 40 cycles at 95 °C (15 s), 60 °C (10 s), and 72 °C (15 s). The homogeneity of qPCR products was confirmed by melting curve analysis (65 °C and a progressive increase up to 95 °C at 0.5 °C/min). Analysis of the data was performed with the CFX Manager™ Software and Microsoft Excel. Calculations were similar as those for telomere length above [42].

### 4.7. RNA Extraction

Total RNA was extracted using a column-based Total RNA Purification Kit (Novazym, Poznań, Poland). Three-zone reagent was added to the frozen powdered brain or liver tissue. After the extraction step, the supernatant was mixed with 70% ethanol, loaded onto a total RNA miniprep column, washed with 70% ethanol, and eluted with ultrapure water. The RNA was DNase treated using the DNase I (Thermo Scientific, Warsaw, Poland). The integrity and quality of the RNA were confirmed by agarose gel electrophoresis and spectroscopy (Thermo Scientific, Warsaw, Poland).

### 4.8. Quantification of Tert and Senescence-Related mRNAs by Real-Time qPCR

cDNA synthesis was carried out in reaction mixtures (20 µL) containing 1 μg of total RNA, RivertAid H Reverse Transcriptase (Thermo Scientific, Warsaw, Poland), and oligo(dT)_23_ (Sigma-Aldrich, Sp z o.o., Poznań, Poland) (42 °C, 60 min and 70 °C, 10 min).

qPCR analyses were carried out in duplicate reaction mixtures (10 µL) containing 20 ng (for *Tert*) or 40 ng (for senescence markers) cDNA, 0.3 µM primers (Supplementary Table S8), and 5 µL iTaq Universal SYBR Green Supermix (Bio-Rad Polska Sp. z o.o.) using a CFX96 Touch Real Time PCR Detection System (Bio-Rad). Cycling parameters were: 95 °C (2 min), 40 cycles at 95 °C (15 s), 60 °C (15 s), and 72 °C (15 s). Following qPCR, the homogeneity of the products was confirmed by melting curve analysis (60 °C and a progressive increase up to 95 °C at 0.5 °C/min). Mouse mRNAs for *Tert*, senescence markers (*p21*, *Il-6*, *Pai-1*, *Mcp1*), and the anti-aging protein Klotho (Kl) were normalized to *β-actin* and *Gapdh* mRNAs. Data analysis was performed with the CFX Manager™ Software, Microsoft Excel, and Statistica. The 2^(−ΔΔ*C*t)^ method was used to calculate relative expression values [42].

### 4.9. Statistical Analyses

Data are expressed as the means ± SEM (Figure 1 and Figure 2) and the means ± SD (Supplementary Tables S1–S6). Comparisons between groups were analyzed using an unpaired 2-sided *t*-test. Associations between telomere length and other variables were analyzed by Pearson’s correlations and linear regression. The interaction effects of age, gender, tHcy, or the *CBS* genotype on TL and mtDNA were examined by analysis of covariance and/or multiple regression models where appropriate. Statistical analysis was performed using Statistica, Version 13 (TIBCO Software Inc., Palo Alto, CA, USA, http://statistica.io).

## Figures and Tables

**Table 1:** Determinants of blood TL in a combined cohort of *Cbs*^−/−^ and *Cbs*^+/−^ mice.

Variable	Pearson Correlation		Multiple Regression			
			Model 1, Hcy		Model 2, *Cbs* Genotype	
	β	*p*	β	*p*	β	*p*
Female (*n* = 39)						
Age *	0.14	0.473	0.15	0.478	0.10	0.559
Hcy ^#^	0.25	0.897	−0.03	0.878		
*Cbs* genotype	−0.28	0.884			−0.08	0.611
			F = 0.27, *p* = 0.768, R^2^ = 0.02		F = 0.36, *p* = 0.698, R^2^ = 0.02	
Male (*n* = 41)						
Age *	−0.56	0.002	−0.50	0.008	−0.38	0.021
Hcy ^#^	−0.34	0.077	−0.16	0.361		
*Cbs* genotype	0.40	0.033			0.11	0.493
			F = 6.40, *p* = 0.006, R^2^ = 0.34		F = 4.01, *p* = 0.027, R^2^ = 0.18	
All (*n* = 80)						
Sex	0.07	0.627	0.03	0.816	0.11	0.774
Age *	−0.28	0.036	−0.24	0.094	−0.190	0.102
Hcy ^#^	−0.17	0.203	−0.08	0.553		
*Cbs* genotype	0.22	0.108			0.04	0.701
			F = 1.6, *p* = 0.195, R^2^ = 0.08		F = 1.6, *p* = 0.193, R^2^ = 0.06	

* Age varied from 44 to 473 days. ^#^ Plasma tHcy varied from 2 to 346 µM.

**Table 2:** Determinants of TL in mouse blood, brain, and liver: multiple regression analysis.

Variable	TL in					
	Blood		Brain		Liver	
	β	*p*	β	*p*	β	*p*
	Female (*n* = 40)		Female (*n* = 20)			
Age *	0.10	0.559	0.53	0.023	0.20	0.392
*Cbs* genotype	−0.08	0.611	−0.10	0.641	0.29	0.228
	F = 0.36, *p* = 0.698, R^2^ = 0.02		F = 3.25, *p* = 0.066, R^2^ = 0.20		F = 1.11, *p* = 0.351, R^2^ = 0.012	
	Male (*n* = 41)		Male (*n* = 23)			
Age *	−0.38	0.021	0.39	0.078	−0.29	0.181
*Cbs* genotype	0.11	0.493	−0.17	0.423	0.18	0.417
	F = 4.01, *p* = 0.027, R^2^ = 0.18				F = 1.65, *p* = 0.217, R^2^ = 0.14	
	All (*n* = 80)		All (*n* = 43)			
Sex	0.11	0.774	−0.55	0.000	0.48	0.001
Age *	−0.190	0.102	0.38	0.003	−0.006	0.964
*Cbs* genotype	0.04	0.701	−0.12	0.340	0.23	0.110
	F = 1.61, *p* = 0.193, R^2^ = 0.06		F = 9.70, *p* = 0.000, R^2^ = 0.39		F = 4.60, *p* = 0.008, R^2^ = 0.26	

* Age varied from 44 to 473 days.

**Table 3:** Determinants of mtDNA in mouse blood, brain, and liver.

Variable	Blood mtDNA *Cbs*^−/−^ (*n* = 40), *Cbs*^+/−^ (*n* = 40)						Brain mtDNA *Cbs*^−/−^ (*n* = 19), *Cbs*^+/−^ (*n* = 24)				Liver mtDNA *Cbs*^−/−^ (*n* = 19), *Cbs*^+/−^ (*n* = 24)			
	Pearson Correlation		Multiple Regression				Pearson Correlation		Multiple Regression		Pearson Correlation		Multiple Regression	
			Model 1, Hcy		Model 2, *Cbs*									
	β	*p*	β	*p*	β	*p*	β	*p*	β	*p*	β	*p*	β	*p*
	Females (*n* = 39)						Females (*n* = 20)				Females (*n* = 20)			
Age *	−0.01	0.960	−0.05	0.832	−0.29	0.090	0.49	0.03	0.49	0.032	0.71	0.000	0.72	0.000
tHcy ^#^	0.08	0.703	0.10	0.670										
*Cbs* genotype	0.02	0.918			0.09	0.600	0.08	0.74	0.10	0.627	0.31	0.177	0.35	0.030
			F = 0.1, *p* = 0.91, R^2^= 0.01		F = 1.8, *p* = 0.18, R^2^ = 0.10				F = 2.2, *p* = 0.09, R^2^ = 0.25				F = 14.1, *p* = 0.000, R^2^ = 0.63	
	Males (*n* = 41)						Males (*n* = 23)				Males (*n* = 23)			
Age *	−0.57	0.005	-0.50	0.023	−0.58	0.000	0.49	0.02	0.49	0.023	0.60	0.003	0.62	0.003
tHcy ^#^	−0.38	0.070	-0.17	0.419										
*Cbs* genotype	0.50	0.014			0.27	0.058	−0.11	0.62	0.000	0.988	−0.04	0.858	0.10	0.580
			F = 5.3, *p* = 0.014, R^2^ = 0.35		F = 13.2, *p* = 0.000, R^2^ = 0.48				F =3.28, *p* = 0.063, R^2^ = 0.24				F = 5.9, *p* = 0.009, R^2^ = 0.37	
	All (*n* = 80)						All (*n* = 43)				All (*n* = 43)			
Sex	−0.33	0.021	−0.36	0.008	−0.25	0.018	−0.40	0.007	−0.41	0.003	−0.11	0.476	−0.10	0.357
Age *	−0.32	0.025	−0.33	0.028	−0.45	0.000	0.42	0.005	0.44	0.002	0.67	0.000	0.71	0.000
tHcy ^#^	−0.21	0.144	−0.08	0.584										
*Cbs* genotype	0.29	0.044			0.18	0.088	0.01	0.97	0.02	0.860	0.14	0.354	0.24	0.041
			F = 4.8, *p* = 0.006, R^2^ = 0.24		F = 9.0, *p* = 0.000, R^2^ = 0.29				F = 7.0, *p* = 0.001, R^2^ = 0.35				F = 14.3, *p* = 0.000, R^2^ = 0.44	

* Age varied from 44 to 473 days. ^#^ Plasma tHcy varied from 2 to 346 µM.

**Table 4:** Determinants of *Pai1*, *p21*, *Mcp-1*, *Il-6*, and *Kl* mRNAs in brains and livers of *Cbs*^−/−^ and *Cbs*^+/−^ mice: multiple regression analysis.

Variable	*Pai-1* mRNA				*p21* mRNA				*Mcp-1* mRNA				*Il-6* mRNA				*Kl* mRNA	
	Brain		Liver		Brain		Liver		Brain		Liver		Brain		Liver		Brain	
	β	*p*	β	*p*	β	*p*	β	*p*	β	*p*	β	*p*	β	*p*	β	*p*	β	*p*
Females (*n* = 18–20)																		
Age *	−0.15	0.524	−0.21	0.362					0.14	0.621	0.23	0.476	0.69	0.001	−0.15	0.497	−0.48	0.043
*Cbs* gene	−0.16	0.520	−0.34	0.021			−0.66	0.007			−0.62	0.006			−0.34	0.016		
TL			−0.11	0.436	0.16	0.536			0.14	0.609								
*Tert*	0.42	0.116	0.71	0.006	−0.10	0.696			−0.05	0.851	0.15	0.006	0.39	0.037	0.62	0.004	0.17	0.443
mtDNA	−0.21	0.419	0.64	0.002			0.04	0.856							0.61	0.002		
	NS		F = 15.7, *p* = 0.000, R^2^ = 0.87		NS		F = 5.4, *p* = 0.02, R^2^ = 0.03		NS		F = 4.84, *p* = 0.015, R^2^=0.49		F = 9.76, *p* = 0.002, R^2^=0.57		F = 20.0, *p* = 0.000, R^2^ = 0.86		F = 3.70, *p* = 0.047, R^2^ = 0.32	
Males (*n* = 20–21)																		
Age *	−0.31	0.207	−0.30	0.327					1.22	0.002	0.52	0.005	0.68	0.039	0.62	0.015	−0.48	0.060
*Cbs* gene	0.31	0.207	−0.30	0.139			−0.47	0.034			−0.44	0.013			−0.14	0.321		
TL					−0.72	0.002	−0.03	0.880	−1.13	0.003			−1.10	0.003				
*Tert*			0.76	0.020	0.11	0.554	−0.50	0.082	0.01	0.960			0.10	0.645	−0.65	0.016	0.18	0.468
mtDNA							0.36	0.189							0.73	0.001		
	F = 4.7, *p* = 0.031, R^2^=0.61		F = 4.5, *p* = 0.020, R^2^ = 0.35		F = 8.78, *p* = 0.004, R^2^ = 0.57		NS		F = 6.17, *p* = 0.010, R^2^ = 0.63		F = 13.0, *p* = 0.000, R^2^ = 0.78		F = 5.19, *p* = 0.016, R^2^ = 0.56		F = 8.9, *p* = 0.000, R^2^ = 0.66		NS	
All (*n* = 39–43)																		
Sex					−0.35	0.044	−0.15	0.270	0.46	0.010								
Age *			-0.22	0.140					0.32	0.061	0.43	0.002	0.45	0.008	0.25	0.093	−0.40	0.011
*Cbs* gene	0.19	0.270	-0.52	0.001			−0.52	0.001			−0.43	0.001			−0.19	0.112		
TL					−0.36	0.028			−0.20	0.259			−0.28	0.095				
*Tert*	0.15	0.378			0.001	0.993							0.38	0.019			0.31	0.044
mtDNA	−0.43	0.018													0.56	0.000		
	F = 3.3, *p* = 0.037, R^2^ = 0.27		F = 7.1, *p* = 0.003, R^2^ = 0.29		F = 6.0, *p* = 0.002, R^2^ = 0.30		F = 8.5, *p* = 0.001, R^2^ = 0.31		F = 3.2, *p* = 0.036, R^2^ = 0.23		F = 13.5, *p* = 0.000, R^2^ = 0.43		F = 4.9, *p* = 0.007, R^2^ = 0.26		F = 16.9, *p* = 0.000, R^2^ = 0.57		F = 8.4, *p* = 0.001, R^2^ = 0.34	

* Mouse age varied from 44 to 473 days.

**Table 5:** Determinants of *p16* and *Il-1β* mRNAs in brains and livers of *Cbs*^−/−^ and *Cbs*^+/−^ mice: multiple regression analysis.

Variable		*p16* mRNA					*Il-1β* mRNA					
		Brain		Liver				Brain		Liver		
		β	*p*		β	*p*		β	*p*		β	*p*
Females (*n* = 18–20)												
Age *		0.84	0.000		0.78	0.085		0.30	0.214		0.34	0.099
*Cbs* gene		0.25	0.065		0.33	0.086		0.14	0.561		0.08	0.616
TL												
*Tert*					−0.07	0.806		−0.18	0.466		−0.38	0.015
mtDNA											0.63	0.008
	F = 23.8, *p* = 0.000, R^2^ = 0.74				F = 7.2, *p* = 0.003, R^2^ = 0.59			NS			F = 11.8, *p* = 0.000, R^2^=0.69	
Males (*n* = 20–21)												
Age *		0.82	0.000		0.41	0.078		0.46	0.142		0.13	0.603
*Cbs* gene		−0.05	0.714		0.04	0.790		0.02	0.939		−0.02	0.876
TL												
*Tert*					0.45	0.052		0.06	0.834		0.08	0.772
mtDNA											0.65	0.004
		F = 20.7, *p* = 0.000, R^2^ = 0.70			F = 12.4, *p* = 0.000, R^2^ = 0.66			NS			F = 7.5, *p* = 0.001, R^2^ = 0.54	
All (*n* = 39–43)												
Sex		0.09	0.014		−0.18	0.180					−0.18	0.140
Age *		0.09	0.000		0.41	0.058					0.17	0.392
*Cbs* gene		0.09	0.114		0.15	0.278		0.03	0.854		−0.02	0.863
TL					−0.01	0.923		0.13	0.419		−0.19	0.184
*Tert*					0.30	0.137		−0.17	0.307		−0.02	0.924
mtDNA					0.04	0.785		0.46	0.005		0.70	0.000
		F = 27.8, *p* = 0.000, R^2^ = 0.69			F = 11.7, *p* = 0.000, R^2^ = 0.56			F = 3.4, *p* = 0.021, R^2^ = 0.31			F = 10.4, *p* = 0.000, R^2^ = 0.64	

* Mouse age varied from 44 to 473 days.

## Supplementary Materials

Supplementary materials can be found at https://www.mdpi.com/1422-0067/21/7/2520/s1. **Figure S1.** Relationships between blood TL, age, and tHcy in *Cbs*^−/−^ and *Cbs* ^+ /−^ mice. Blood TL vs. age in (**A**) female and (**B**) male mice. (**C**) Blood TL vs. tHcy; **Figure S2.** Telomere length (TL) in brains (**A**) and livers (**B**) of *Cbs***^−/−^** and *Cbs*^+/−^ mice. TL was quantified by qPCR in *Cbs***^−/−^** mice (*n* = 21) and sex- and age-matched control *Cbs*^+/−^ siblings (*n* = 22) as described in the Materials and Methods. Box and whiskers represent the mean ± SEM and the mean ± 95% CI, respectively; **Figure S3.** *Tert* mRNA levels in brains (**A**) and livers (**B**) of *Cbs***^−/−^** and *Cbs*^+/−^ mice. *Tert* mRNA was quantified by qPCR in *Cbs***^−/−^** mice (*n* = 21) and sex- and age-matched control *Cbs*^+/−^ siblings (*n* = 22) as described in the Materials and Methods. Box and whiskers represent the mean ± SEM and the mean ± 95% CI, respectively; **Figure S4.** mtDNA levels in blood (**A**), livers (**B**), and brains (**C**) of *Cbs***^−/−^** and *Cbs*^+/−^ mice. mtDNA was quantified by qPCR in *Cbs***^−/−^** mice (blood, *n* = 40; liver and brain, *n* = 21) and sex- and age-matched control *Cbs*^+/−^ siblings (blood, *n* = 40; liver and brain, *n* = 22) as described in the Materials and Methods. Box and whiskers represent the mean ± SEM and the mean ± 95% CI, respectively; **Table S1.** Aging/senescence-related mRNAs in the liver of *Cbs*^−/−^ and *Cbs*^+/−^ mice stratified by sex and age; **Table S2.** Aging/senescence-related mRNAs in the brain of *Cbs*^−/−^ and *Cbs*^+/-^ mice stratified by sex and age; **Table S3.** Blood TL, plasma tHcy levels in *Cbs*^−/−^ and *Cbs*^+/−^ mice stratified by sex and age; **Table S4.** Brain TL and *Tert* mRNA expression in *Cbs*^−/−^ and *Cbs*^+/−^ mice stratified by sex and age; **Table S5.** Liver TL and *Tert* mRNA expression in *Cbs*^−/−^ and *Cbs*^+/−^ mice stratified by sex and age; **Table S6.** mtDNA levels in the blood, brain, and liver of *Cbs*^−/−^ and *Cbs*^+/−^ mice stratified by sex and age; **Table S7.** Determinants of *Tert* mRNA in the brain and liver of *Cbs*^−/−^ and *Cbs*^+/−^ mice: Multiple regression analysis; **Table S8.** List of primers used for mouse mRNA quantification by RT-qPCR.
