# Supplementary material for: The Cbs Locus Affects the Expression of Senescence Markers and mtDNA Copy Number, but not Telomere Dynamics in Mice
Source: Int J Mol Sci. 2020 Apr 5;21(7):2520. doi: 10.3390/ijms21072520 (PMC7177707; doi:10.3390/ijms21072520)
Supplement: Supplementary file 1 [file ijms-21-02520-s001.pdf]

Supplementary Material

Supplementary Figure S1.
Supplementary Figure S2.
Supplementary Figure S3.
Supplementary Figure S4.
Supplementary Table S1.
Supplementary Table S2.
Supplementary Table S3.
Supplementary Table S4.
Supplementary Table S5.
Supplementary Table S6.
Supplementary Table S7.
Supplementary Table S8.

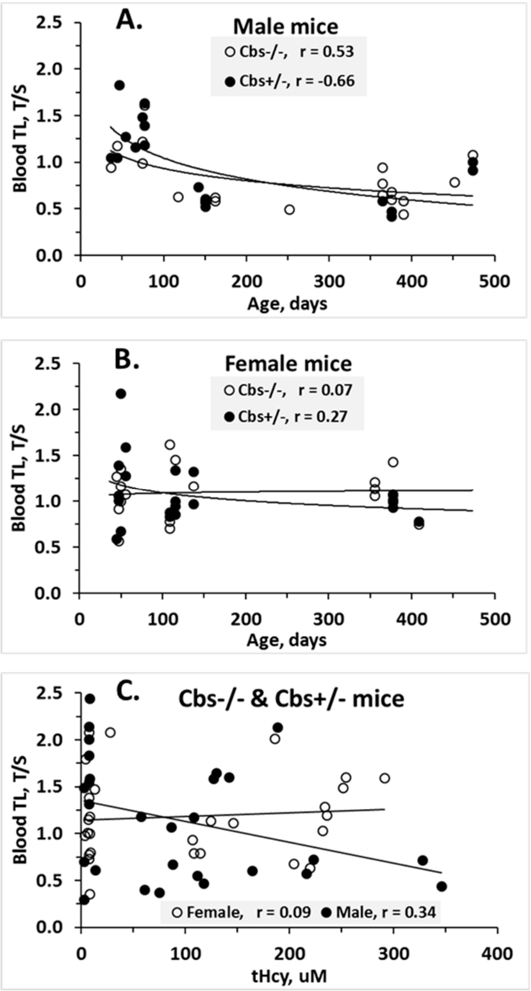

Supplementary Figure S1. Relationships between blood TL, age, and tHcy in *Cbs*$^{-/-}$ and *Cbs* $^{+/-}$ mice. Blood TL vs. age in (**A**) female and (**B**) male mice. (**C**) Blood TL vs. tHcy.

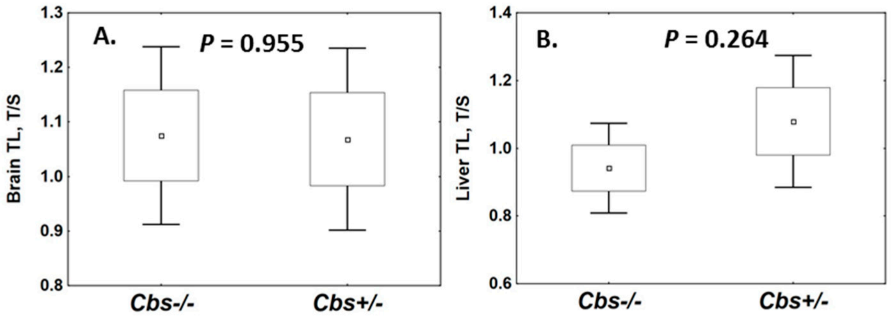

Supplementary Figure S2. Telomere length (TL) in brains (**A**) and livers (**B**) of *Cbs*$^{-/-}$ and *Cbs*$^{+/-}$ mice. TL was quantified by qPCR in *Cbs*$^{-/-}$ mice (n = 21) and sex- and age-matched control *Cbs*$^{+/-}$ siblings (n = 22) as described in the Materials and Methods. Box and whiskers represent the mean ± SEM and the mean ± 95% CI, respectively.

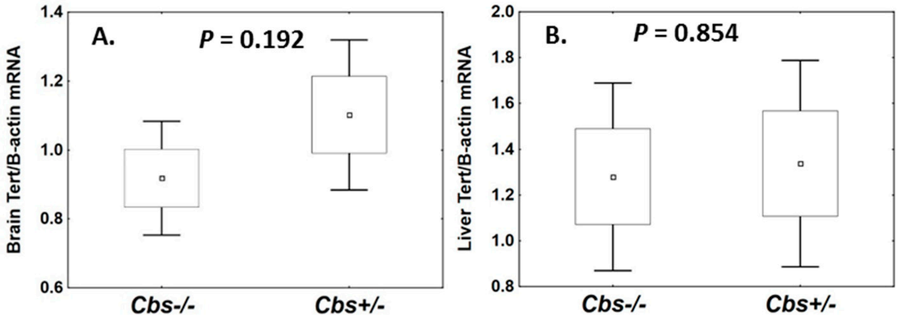

Supplementary Figure S3. *Tert* mRNA levels in brains (**A**) and livers (**B**) of *Cbs*$^{-/-}$ and *Cbs*$^{+/-}$ mice. *Tert* mRNA was quantified by qPCR in *Cbs*$^{-/-}$ mice (n = 21) and sex- and age-matched control *Cbs*$^{+/-}$ siblings (n = 22) as described in the Materials and Methods. Box and whiskers represent the mean ± SEM and the mean ± 95% CI, respectively.

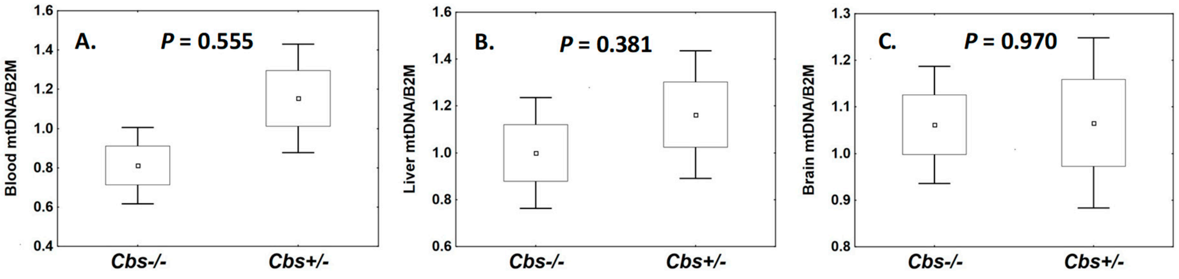

Supplementary Figure S4. mtDNA levels in blood (**A**), livers (**B**), and brains (**C**) of *Cbs*$^{-/-}$ and *Cbs*$^{+/-}$ mice. mtDNA was quantified by qPCR in *Cbs*$^{-/-}$ mice (blood, n = 40; liver and brain, n = 21) and sex- and age-matched control *Cbs*$^{+/-}$ siblings (blood, n = 40; liver and brain, n = 22) as described in the Materials and Methods. Box and whiskers represent the mean ± SEM and the mean ± 95% CI, respectively.

Supplementary Table S1. Aging/senescence-related mRNAs in the liver of *Cbs*$^{-/-}$ and *Cbs*$^{+/-}$ mice stratified by sex and age.

**Genotype (*n*)**	**Sex (*n*)**	**Age***	***Pai-1*/β-*actin* mRNA**			***p21*/β-*actin* mRNA**			***Mcp1*/β-*actin* mRNA**			***Il-6*/β-*actin* mRNA**		
			**Mean ± SD (*n*)**	**FC#; $P_{genotype}$**	**P_{sex}**	**Mean ± SD (*n*)**	**FC#; $P_{genotype}$**	**FC#; P_{sex}**	**Mean ± SD (*n*)**	FC#; $P_{genotype}$	FC#; P_{sex}	Mean ± SD (*n*)	FC#; $P_{genotype}$	FC#; P_{sex}
Cbs$^{-/-}$ (18–20)	♀ (9–10)	Young	10.7 ± 6.4 (4)	10.7; **0.025**	13.4; 0.060	5.3 ± 3.2 (4)	7.6; **0.016**	0.87; 0.272	4.2 ± 2.2 (4)	11.0; **0.014**	2.0; 0.202	0.78 ± 1.11 (3)	4.6; 0.311	0.55; 0.500
		Old	3.1 ± 1.5 (6)	4.4; **0.006**	1.47; 0.318	6.3 ± 4.8 (6)	4.2; 0.059	1.75; 0.124	4.3 ± 1.8 (6)	1.4; 0.174	0.34; **0.004**	2.87 ± 1.50 (6)	0.8; 0.525	0.71; 0.252
		FC#; P_{age}	0.29; **0.021**			1.2; 0.589			1.0; 0.906			1.2; 0.072		
	♂ (8–10)	Young	1.3 ± 1.3 (3)	0.8; 0.571		10.4 ± 7.5 (2)	8.7; **0.005**		2.1 ± 1.0 (3)	3.0; **0.005**		1.43 ± 1.05 (3)	8.4; **0.063**	
		Old	4.5 ± 2.9 (5)	3.0; 0.106		3.1 ± 2.8 (6)	2.4; 0.123		12.7 ± 5.3 (6)	3.2; **0.034**		4.06 ± 1.98 (7)	1.0; 0.923	
		FC#;P_{age}	3.5; 0.132			0.30; 0.065			6.0; **0.013**			3.0; 0.066		
Cbs$^{+/-}$ (20–23)	♀ (9)	Young	1.0 ± 0.9 (4)		0.59; 0.230	0.70 ± 0.37 (5)		0.58;0.124	0.38 ± 0.12 (4)		0.49; 0.307	0.17 ± 0.10 (4)		0.29; **0.010**
		Old	0.7 ± 0.2 (5)		0.45; 0.216	1.50 ± 0.62 (4)		1.15;0.676	3.12 ± 0.39 (5)		0.69; 0.715	3.42 ± 1.29 (5)		0.88; 0.796
		FC#; P_{age}	0.7; 0.384			2.1; **0.047**			8.2; **0.000**			20.1; **0.002**		
	♂ (10–14)	Young	1.7 ± 0.8 (7)			1.2 ± 0.5 (7)			0.78 ± 0.62 (7)			0.59 ± 0.24 (7)		
		Old	1.5 ± 1.4 (4)			1.3 ± 0.9 (7)			1.66 ± 1.58 (4)			3.90 ± 3.72 (6)		
		FC#; P_{age}	0.9; 0.784			1.1; 0.749			2.1; 0.217			6.6; **0.037**		

* Young = 63- to 66-day-old; Old = 354- to 408-day-old. # FC, fold change.

Supplementary Table S2. Aging/senescence-related mRNAs in the brain of *Cbs*$^{-/-}$ and *Cbs* $^{+/-}$ mice stratified by sex and age.

Genotype (*n*)	Sex (*n*)	Age*	*Pai-1*/β-*actin* mRNA Mean ± SD (*n*)	FC[#]; P_{gen}	P_{sex}	*p21*/β-*actin* mRNA Mean ± SD (*n*)	FC[#]; P_{gen}	FC[#]; P_{sex}	*Mcp1*/β-*actin* mRNA Mean ± SD (*n*)	FC[#]; P_{gen}	P_{sex}	*Il-6*/β-*actin* mRNA Mean ± SD (*n*)	FC[#]; P_{gen}	FC[#]; P_{sex}	*Kl*/β-*actin* mRNA Mean ± SD (*n*)	FC[#]; P_{gen}	FC[#]; P_{sex}
Cbs$^{-/-}$ (17–21)	♀ (8–11)	Young	1.38 ± 0.14 (3)	1.3; 0.404	0.167	1.87 ± 0.63 (4)	**1.9**; **0.035**	0.206	0.66 ± 0.12 (3)	0.7; 0.259	0.266	1.54 ± 1.24 (4)	2.0; 0.286	1.9; 0.383	2.00 ± 0.34 (4)	1.4; 0.192	**2.5**; **0.010**
		Old	0.97 ± 0.33 (5)	0.9; 0.361	0.623	1.83 ± 0.77 (7)	0.360	**2.9**; **0.004**	1.03 ± 0.21 (7)	**1.4**; **0.020**	0.622	1.34 ± 0.29 (7)	0.83; 0.417	**1.6** **0.032**	1.17 ± 0.56 (7)	1.4; 0.996	0.168
		FC[#]; P_{age}	0.7; 0.093			1.0; 0.936			1.6; **0.02**			0.9; 0.678			**0.6**; **0.022**		
	♂ (8–10)	Young	0.76 ± 0.60 (2)	0.5; 0.366		1.09 ± 0.80 (3)	0.750		0.89 ± 0.29 (3)	0.8; 0.421		0.81 ± 0.45 (3)	0.71; 0.520		0.80 ± 0.46 (3)	0.53; 0.138	
		Old	0.71 ± 0.54 (7)	0.7; 0.411		0.64 ± 0.46 (7)	0.818		1.16 ± 0.68 (5)	0.8; 0.773		0.83 ± 0.47 (7)	0.83; 0.495		0.80 ± 0.41 (7)	0.99; 0.465	
		P_{age}	0.911			0.286			0.547			0.949			0.996		
Cbs$^{+/-}$ (19–22)	♀ (8–9)	Young	1.03 ± 0.63 (4)		0.527	0.96 ± 0.23 (4)		0.254	0.95 ± 0.37 (4)		0.599	0.77 ± 0.46 (4)		0.270	1.44 ± 0.69 (4)		0.878
		Old	1.05 ± 0.29 (5)		0.989	1.43 ± 0.66 (5)		**2.1**; **0.036**	0.72 ± 0.08 (4)		0.052	1.61 ± 0.77 (5)		0.121	0.83 ± 0.28 (4)		0.947
		P_{age}	0.951			0.231			0.273			0.098			0.156		
	♂ (10–13)	Young	1.42 ± 1.04 (6)			1.41 ± 0.69 (7)			1.07 ± 0.27 (6)			1.14 ± 0.53 (7)			1.50 ± 0.65 (7)		
		Old	1.05 ± 0.55 (4)			0.69 ± 0.28 (6)			1.41 ± 0.54 (6)			1.00 ± 0.37 (6)			0.81 ± 0.54 (6)		
		FC[#]; P_{age}	0.7; 0.535			**0.5**; **0.038**			1.3; 0.238			0.9; 0.596			0.5; 0.064		

* Young = 63- to 66-day-old; Old = 354- to 408-day-old. [#] FC, fold change.

Supplementary Table S3. Blood TL, plasma tHcy levels in *Cbs*$^{-/-}$ and *Cbs*$^{+/-}$ mice stratified by sex and age.

Genotype (*n*)	Sex (*n*)	Age* (*n*)	tHcy, µM			TL, T/S		
			Mean ± SD	$P_{genotype}$	P_{sex}	Mean ± SD	$P_{genotype}$	P_{sex}
Cbs$^{-/-}$ (40)	♀ (20)	Young (12)	133 ± 64	**<0.000**	0.990	1.08 ± 0.31	0.820	0.507
		Old (8)	238 ± 33	**<0.000**	**0.044**	1.10 ± 0.19	0.374	**<0.000**
		P_{age}	**0.002**			0.855		
	♂ (20)	Young (6)	133 ± 42	**<0.000**		1.19 ± 0.27	0.335	
		Old (14)	156 ± 96	**<0.000**		0.68 ± 0.17	0.615	
		P_{age}	0.531			**<0.000**		
Cbs$^{+/-}$(40)	♀ (20)	Young (14)	6.4 ± 1.8		0.180	1.12 ± 0.41		0.165
		Old (6)	8.7 ± 2.8		0.669	1.01 ± 0.18		**0.002**
		P_{age}	0.096			0.564		
	♂ (20)	Young (10)	7.3 ± 0.2			1.34 ± 0.27		
		Old (10)	5.1 ± 5.4			0.64 ± 0.19		
		P_{age}	0.272			**<0.000**		

* Young = 36- to 115-day-old; Old = 117- to 473-day-old; ♀ *Cbs*$^{-/-}$, ♂ *Cbs*$^{-/-}$, ♀ *Cbs* $^{+/-}$ and ♂ *Cbs*$^{+/-}$.

Supplementary Table S4. Brain TL and *Tert* mRNA expression in *Cbs*$^{-/-}$ and *Cbs*$^{+/-}$ mice stratified by sex and age.

Genotype (*n*)	Sex (*n*)	Age*	Rel. brain TL, T/S			Rel. brain Tert/β-actin mRNA		
			Mean ± SD	$P_{genotype}$	P_{sex}	Mean ± SD	$P_{genotype}$	P_{sex}
Cbs$^{-/-}$ (19)	♀ (10)	young	0.73 ± 0.09	0.527	0.159	1.29 ± 0.33	0.916	**0.017**
		old	0.95 ± 0.22	0.948	**0.010**	0.87 ± 0.35	0.275	0.352
		P_{age}	0.095			0.154		
	♂ (9)	young	0.94 ± 0.22	0.504		0.55 ± 0.17	**0.035**	
		old	1.53 ± 0.34	0.155		0.78 ± 0.23	0.423	
		P_{age}	0.063			0.184		
Cbs$^{+/-}$ (24)	♀ (10)	young	1.00 ± 0.78		0.746	1.27 ± 0.32		0.978
		old	0.96 ± 0.32		0.273	1.28 ± 0.61		0.067
		P_{age}	0.916			0.977		
	♂ (14)	young	1.11 ± 0.24			1.26 ± 0.39		
		old	1.17 ± 0.26			0.66 ± 0.21		
		P_{age}	0.702			**0.022**		

* Young = 63- to 66-day-old; Old = 354- to 408-day-old.

Supplementary Table S5. Liver TL and *Tert* mRNA expression in *Cbs*$^{-/-}$ and *Cbs*$^{+/-}$ mice stratified by sex and age.

Genotype (*n*)	Sex (*n*)	Age*	Rel. liver TL, T/S			Rel. liver *Tert*/β-actin mRNA		
			**Mean ± SD**	$P_{genotype}$	P_{sex}	**Mean ± SD**	$P_{genotype}$	P_{sex}
Cbs$^{-/-}$ (19)	♀ (10)	young	1.08 ± 0.36	0.909	0.353	0.24 ± 0.10	0.264	**0.020**
		old	1.11 ± 0.30	0.186	**0.014**	2.22 ± 0.81	0.512	**0.029**
		P_{age}	0.892			**0.001**		
	♂ (9)	young	0.81 ± 0.35	0.415		0.43 ± 0.03	0.859	
		old	0.75 ± 0.19	0.735		1.43 ± 0.47	0.428	
		P_{age}	0.737			**0.008**		
Cbs$^{+/-}$ (24)	♀ (10)	young	1.12 ± 0.47		0.681	0.90 ± 0.79		0.318
		old	1.50 ± 0.58		**0.018**	2.89 ± 1.07		**0.012**
		P_{age}	0.325			**0.010**		
	♂ (14)	young	1.01 ± 0.40			0.56 ± 0.14		
		old	0.78 ± 0.23			1.43 ± 0.39		
		P_{age}	0.228			**0.000**		

* Young = 63- to 66-day-old; Old = 354- to 408-day-old.

Supplementary Table S6. mtDNA levels in the blood, brain, and liver of *Cbs*$^{-/-}$ and *Cbs*$^{+/-}$ mice stratified by sex and age.

**Genotype (*n*)**	**Sex (*n*)**	**Age***	**Rel. blood mtDNA/*B2M* DNA**			**Rel. brain mtDNA/*B2M* DNA**			**Rel. liver mtDNA/*B2M* DNA**		
			**Mean ± SD (*n*)**	**FC; $P_{genotype}$**	**P_{sex}**	**Mean ± SD (*n*)**	**FC; $P_{genotype}$**	**P_{sex}**	**Mean ± SD (*n*)**	**FC; $P_{genotype}$**	**P_{sex}**
Cbs$^{-/-}$ (21–39)	♀ (11–19)	Young	1.39 ± 0.78 (8)	0.9; 0.861	0.322	0.73 ± 0.08 (4)	0.9; 0.269	**0.045**	0.44 ± 0.29 (4)	0.7; 0.321	0.250
		Old	0.68 ± 0.13 (11)	0.8; 0.139	0.613	0.98 ± 0.23 (7)	1.0; 0.916	**0.002**	1.12 ± 0.49 (7)	0.6; 0.059	0.525
		P_{age}	**0.004**			0.062			**0.036**		
	♂ (10–20)	Young	1.51 ± 1.09 (6)	0.7; 0.118		0.95 ± 0.14 (3)	1.1; 0.514		0.75 ± 0.32 (3)	1.1; 0.897	
		Old	0.76 ± 0.40 (14)	0.9; 0.641		1.38 ± 0.14 (7)	0.94; 0.922		1.31 ± 0.58 (7)	0.8; 0.426	
		P_{age}	**0.046**			**0.002**			0.160		
Cbs$^{+/-}$ (22–38)	♀ (9–20)	Young	1.46 ± 0.72 (14)		**0.041**	0.84 ± 0.17 (4)		0.561	0.68 ± 0.25 (4)		0.858
		Old	0.84 ± 0.75 (6)		0.927	1.00 ± 0.38 (5)		0.264	1.75 ± 0.53 (5)		0.620
		P_{age}	0.103			0.463			**0.004**		
	♂ (13–18)	Young	2.15 ± 0.96 (10)			0.89 ± 0.13 (7)			0.68 ± 0.22 (7)		
		Old	0.87 ± 0.62 (8)			1.40 ± 0.60 (7)			1.58 ± 0.69 (6)		
		P_{age}	**0.003**			**0.048**			**0.004**		

* Young = 44 to 77-day-old; Old = 108–390-day-old. * Young = 63 to 66-day-old; Old = 354 to 408-day-old.

Supplementary Table S7. Determinants of *Tert* mRNA in the brain and liver of *Cbs*$^{-/-}$ and *Cbs*$^{+/-}$ mice: Multiple regression analysis.

Variable	*Tert* mRNA			
	**Brain**		**Liver**	
	β	*p*	β	*p*
Female (*n* = 19)				
Age*	-0.18	0.438	**0.81**	**0.000**
Cbs genotype	0.23	0.343	0.08	0.531
TL	0.08	0.765	**0.34**	**0.015**
	F = 0.83, p = 0.451, R^2 = 0.09		**F = 25.6, p = 0.000, R^2 = 0.76**	
Male (*n* = 22)				
Age*	−0.31	0.207	**0.84**	**0.000**
Cbs genotype	0.41	0.100	0.02	0.908
TL	0.15	0.716	0.13	0.380
	F = 1. 9, p = 0.187, R^2 = 0.21		**F = 18.1, p = 0.003, R^2 = 0.61**	
All (*n* = 41)				
Sex	**−0.43**	**0.006**	-0.13	0.194
Age*	−0.22	0.152	**0.72**	**0.000**
Cbs genotype	0.24	0.115	0.07	0.484
TL	0.05	0.796	**0.37**	**0.000**
	**F = 4.3, p = 0.011, R^2 = 0.28**		**F = 28.9, p = 0.000, R^2 = 0.69**	

*Mouse age varied from 63 to 408 days.

Supplementary Table S8. List of primers used for mouse mRNA quantification by RT-qPCR.

Gene name	Forward 5′→3′	Reverse 5′→3′
Tert	GTGAACAGCCTCCAGACAG	TTCCTAACACGCTGGTCAAA
Pai-1	ACGCCTGGTGCTGGTGAATGC	ACGGTGCTGCCATCAGACTTGTG
Mcp1	GGGATCATCTTGCTGGTGAA	AGGTCCCTGTCATGCTTCTG
p21	GGCAGACCAGCCTGACAGAT	TTCAGGGTTTTCTCTTGCAGAAG
Il-6	TGGTACTCCAGAAGACCAGAGG	AACGATGATGCACTTGCAGA
Kl	TGTATGTGACAGCCAATGGAATCG	GAATACGCAAAGTAGCCACAAAGG
p16	GAACTCTTTCGGTCGTACCC	CGAATCTGCACCGTAGTTGA
Il-1β	CAACCAACAAGTGATATTCTCCATG	GATCCACACTCTCCAGCTGCA
β-actin	TGTTACCAACTGGGACGACA	GGGGTGTTGAAGGTCTCAAA
Gapdh	CGTCCCGTAGACAAAATGGT	TTGATGGCAACAATCTCCAC
